# Lipidomic analysis reveals drug-induced lipoxin synthesis in glaucoma treatment

**DOI:** 10.1172/jci.insight.192010

**Published:** 2026-02-24

**Authors:** David J. Mathew, Shubham Maurya, Julian Ho, Izhar Livne-Bar, Darren Chan, Jenny Wanyu Zhang, Yvonne M. Buys, Marisa Sit, Graham Trope, Donna M. Peters, John G. Flanagan, Karsten Gronert, Jeremy M. Sivak

**Affiliations:** 1 Donald K. Johnson Eye Institute, Krembil Research Institute, University Health Network, Toronto, Canada.; 2 Department of Ophthalmology and Vision Science, and; 3 Department of Laboratory Medicine and Pathobiology, University of Toronto School of Medicine, Toronto, Canada.; 4 Herbert Wertheim School of Optometry & Vision Science, and; 5 Vision Science Program, University of California Berkeley, Berkeley, California, USA.; 6 Pathology and Laboratory Medicine, and; 7 Ophthalmology and Visual Sciences, University of Wisconsin School of Medicine and Public Health, Madison, Wisconsin, USA.; 8 Infectious Disease and Immunity Program, University of California Berkeley, Berkeley, California, USA.

**Keywords:** Inflammation, Ophthalmology, Eicosanoids, Lipidomics, Pharmacology

## Abstract

Synthetic prostaglandin analogs, such as latanoprost, are first-line treatments to reduce intraocular pressure (IOP) in the management of glaucoma, treating millions of patients daily. Glaucoma is a leading cause of blindness, characterized by progressive optic neuropathy, with elevated IOP being the sole modifiable risk factor. Despite this importance, the underlying latanoprost mechanism of action is still not well defined, being associated with both acute and long-term activities, and a growing list of ocular side effects. Prostaglandins are eicosanoid lipid mediators. Yet, there has not been a comprehensive assessment of small lipid mediators in glaucomatous eyes. Here, we performed a lipidomic screen of aqueous humor sampled from patients with glaucoma and healthy control eyes. The resulting signature was surprisingly focused on significantly elevated levels of arachidonic acid (AA) and its derivative, the antiinflammatory and cytoprotective mediator, lipoxin A_4_ (LXA_4_), in glaucomatous eyes. Subsequent experiments revealed that this response was drug induced, due to latanoprost actions on trabecular meshwork cells, rather than a consequence of elevated IOP. We demonstrate that increased LXA_4_ inhibited proinflammatory cues and promoted TGF-β production in the anterior chamber. In concert, an autocrine prostaglandin circuit mediated canonical rapid IOP lowering. This work reveals parallel mechanisms underlying acute and long-term latanoprost activities during glaucoma treatment.

## Abstract 2

New lipidomic evidence reveals Latanoprost induces production of Lipoxin A4 in glaucoma patients to inhibit pro-inflammatory cues and promote TGF-beta production.

## Graphical abstract

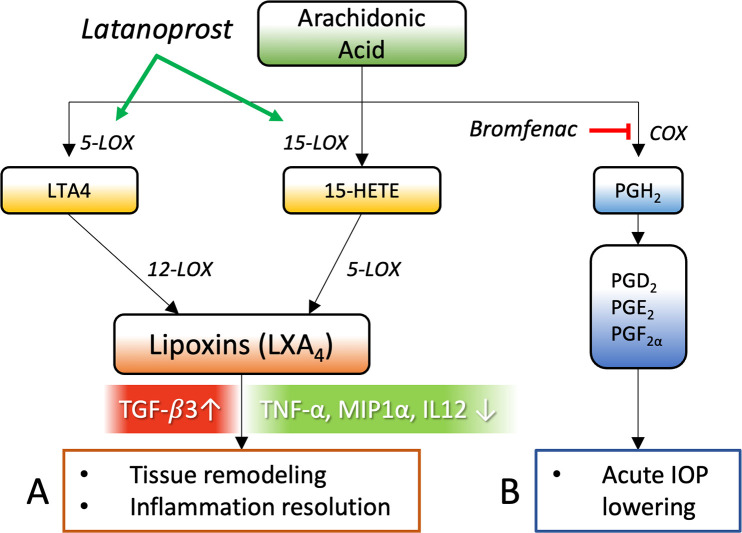

## Introduction

Glaucoma represents a spectrum of diseases characterized by progressive retinal ganglion cell degeneration, optic neuropathy, and visual field loss, with elevated intraocular pressure (IOP) being the sole modifiable risk factor (1, 2). Glaucoma is a leading cause of irreversible blindness and is estimated to affect over 110 million people by 2040 (3). Reduction of IOP is the standard of care in glaucoma treatment and is achieved using medical, laser, or surgical management. These approaches either increase the outflow of aqueous fluid from the anterior chamber of the eye or reduce fluid production. However, the relationship between IOP and glaucoma progression is complex. For example, a lowered mean IOP is not always a reliable indicator of disease stability, and increased risk of glaucoma progression is associated with a higher diurnal variation in IOP (4). In addition, the risk of disease progression is higher for the same IOP in more advanced stages of glaucoma (5). Therefore, it is critical to uncover additional biochemical mediators driving glaucoma pathogenesis and their links to IOP regulation.

The most prescribed class of IOP-lowering medications are prostaglandin analogs, followed by β-adrenergic blockers, carbonic anhydrase inhibitors, and α-2-adrenergic agonists. Prostaglandin analogs (PGAs), such as latanoprost, bimatoprost, and travoprost are the most commonly used first-line agents for medical management (6–8), treating millions of patients with glaucoma daily (9). These drugs increase both trabecular and uveoscleral aqueous humor outflow (10, 11) through 2 proposed mechanisms: prostaglandin F (FP) receptor–mediated ciliary muscle relaxation (12) and increased permeability of outflow tissues via TGF-β–mediated matrix metalloproteinase (MMP) activity (13–16). Despite their widespread use, the detailed biochemical mechanisms linking PGA actions to these dual IOP effects remain poorly understood, particularly the explanation behind recently reported long-term IOP lowering after cessation of treatment (14, 17, 18). Although generally well tolerated, long-term PGA treatment is accompanied by diverse and well-documented ocular and periocular adverse side effects, such as changes to eyelid and iris pigmentation, hyperemia, iritis, corneal thinning, eyelash growth, periorbital fat atrophy, and potential associations with macular edema and uveitis (19–21). Therefore, it has become increasingly important to unravel the underlying drug mechanisms in order to potentially uncouple and separately target these activities.

Endogenous prostaglandins are lipid mediators with key roles in normal physiology and drive or amplify inflammatory responses. They are enzymatically generated from arachidonic acid (AA) by cyclooxygenases (COX-1 and COX-2) (22). Prostaglandins are part of an intrinsic eicosanoid (AA metabolite) network in tissues that also includes bioactive lipoxygenase (LOX) metabolites, such as the lipoxins that are produced by the interaction of 5-LOX and 12/15-LOX (23). In contrast to prostaglandins, lipoxins A_4_ and B_4_ (LXA_4_ and LXB_4_) are themselves potent mediators inhibiting and resolving inflammation and maintaining cellular homeostasis (24–26). LXA_4_ signaling and production has been linked to a variety of ocular surface and inflammatory diseases (26, 27). We also recently demonstrated that therapeutic lipoxin supplementation results in structural and functional neuronal rescue in rodent glaucoma models (28–30), and reduced neuroinflammation (26, 31, 32). Interestingly, formation of lipoxins, or related specialized proresolving mediators (SPMs), can also be triggered pharmacologically, for example by statins or aspirin acetylation of COX-2 (33–39). Surprisingly, despite these central roles, the production of these and other small lipid mediators has not been well studied in patients with glaucoma.

Given this background of clinical pharmacology, shared substrates, and potential interactions between prostaglandins and other lipid mediator circuits, we decided to profile these signals in patients with glaucoma. Here, we present a metabolomic characterization of LOX- and COX-generated mediators in aqueous humor sampled from patients with glaucoma compared with healthy controls. Unexpectedly, the resulting glaucoma signature was tightly focused on significantly elevated levels of AA and LXA_4_ in patients. Our subsequent experiments investigated the regulation of this AA/LXA_4_ circuit to reveal insights into latanoprost’s mechanism of action.

## Results

### The AA/lipoxin pathway is specifically elevated in glaucomatous aqueous humor.

**Figure 1:**
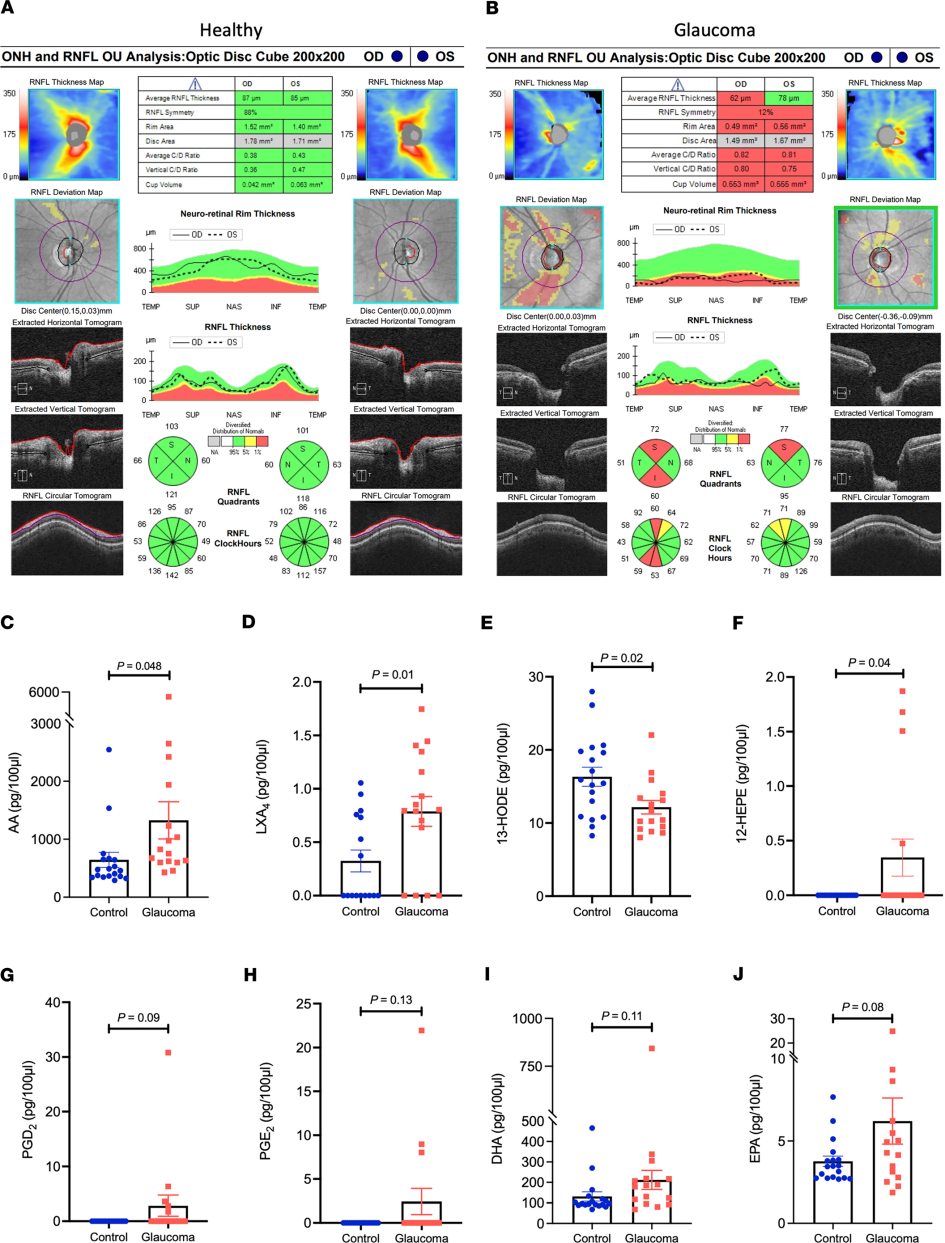
The AA/lipoxin pathway is specifically elevated in glaucomatous aqueous humor. (**A**) Representative optical coherence tomography (OCT)scans from a control patient showing healthy RNFL and optic nerve head (ONH) in both eyes. (**B**) Representative OCT scans from a glaucomatous patient showing significant superior and inferior RNFL thinning in the right eye and superior RNFL thinning in the left eye. (**C**–**F**) Lipidomic analysis of mediators and metabolites from glaucomatous and healthy aqueous humor showed significantly elevated concentrations of (**C**) AA and (**D**) LXA_4_. (**E**) In comparison, 13-HODE was detected at significantly lower levels in glaucomatous aqueous humor. (**F**) 12-HEPE levels were significantly elevated in the glaucoma group, although statistically driven by only 4 samples (*P* values are indicated. Data are presented as mean ± SEM). (**G**–**J**) Concentrations of additional analytes detected in human aqueous humor samples included (**G**) PGD_2_, (**H**) PGE_2_, (**I**) DHA, and (**J**) EPA. However, none of these differences reached statistical significance. For all charts, *P* values are indicated by 2-tailed, unpaired *t* tests, and data are presented as mean ± SEM. OD, right eye; OS, left eye; RNFL, retinal nerve fiber layer.

Aqueous humor samples collected from patients with primary open angle glaucoma (POAG), or matched controls, were analyzed by targeted LC-MS/MS–based lipidomics. Fifteen patients were enrolled in each group (providing 16 and 18 eye samples in the glaucoma and control groups, respectively). There was no statistically significant difference in age or sex between the 2 groups (*P* = 0.25 and 0.30, respectively; Table 1). Most patients in the glaucoma group had advanced disease, reflected in a significantly increased cup-to-disc ratio and reduced retinal nerve fiber layer (RNFL) thickness compared with control patient samples (Table 1 and Figure 1, A and B).

Lipidomic analysis focused on LOX and COX pathways and their polyunsaturated fatty acid (PUFA) substrates. The analysis included eicosanoids (prostaglandins, leukotrienes, and lipoxins), AA and other PUFAs, pathway markers and metabolites, and select ω-3 PUFA–derived SPMs (see Supplemental Table 1 for a full list of these results; supplemental material available online with this article; https://doi.org/10.1172/jci.insight.192010DS1).

Four mediators showed striking changes in concentration between groups. Most significant were the substrate AA (643.07 ± 127.15 vs. 1328.04 ± 312.43 pg/100 μL of aqueous humor, *P* = 0.048) and the AA LOX product LXA_4_ (0.74 ± 0.08 vs. 1.05 ± 0.36 pg/100 μL of aqueous humor, *P* = 0.01), whose concentrations were strongly elevated in the aqueous humor of patients with glaucoma (Figure 1, C and D). In addition, 13-hydroxyoctadecadienoic acid (13-HODE), a product of linoleic acid metabolism by 15-LOX, was present at significantly lower levels in glaucomatous aqueous humor (Figure 1E). The levels of 12-hydroxyeicosapentaenoic acid (12-HEPE), an eicosapentaenoic acid–derived (EPA-derived) metabolite, were also significantly higher in glaucomatous aqueous humor (1.38 ± 0.62 vs. 0 pg/100 μL, *P* = 0.04), but this result was less convincingly driven by changes in only a few samples (Figure 1F). In addition, the AA-derived prostaglandin E_2_ (PGE_2_) and prostaglandin D_2_ (PGD_2_) were elevated in some glaucoma samples, as well as the ω-3 PUFAs docosahexaenoic acid (DHA) and EPA, but these changes did not reach significance between the groups (Figure 1, G–J).

No other PUFA, LOX, or COX metabolites were identified at significant levels by our LC-MS/MS method (Supplemental Table 1). Therefore, these results suggest a select response in the activity of AA and LXA_4_ circuits that generates a marked increase in their levels in glaucomatous aqueous humor. Formation and increased levels of LXA_4_ are consistent with immunofluorescent analysis of anterior segment outflow tissues from healthy human donor eyes and glaucomatous patient eyes. Ciliary muscle, vasculature, and outflow tissues demonstrated consistent staining for the LXA_4_ biosynthetic enzymes 5-LOX and 15-LOX (Supplemental Figure 3). Interestingly, matched sections from 2 glaucomatous patient eyes showed prominently increased 5-LOX staining and mildly increased 15-LOX staining in the trabecular meshwork (TM) (Supplemental Figure 3). Therefore, we focused on the TM and outflow tissues in follow-up experiments.

### The AA/lipoxin circuit is induced by latanoprost treatment in human TM cells.

**Figure 2:**
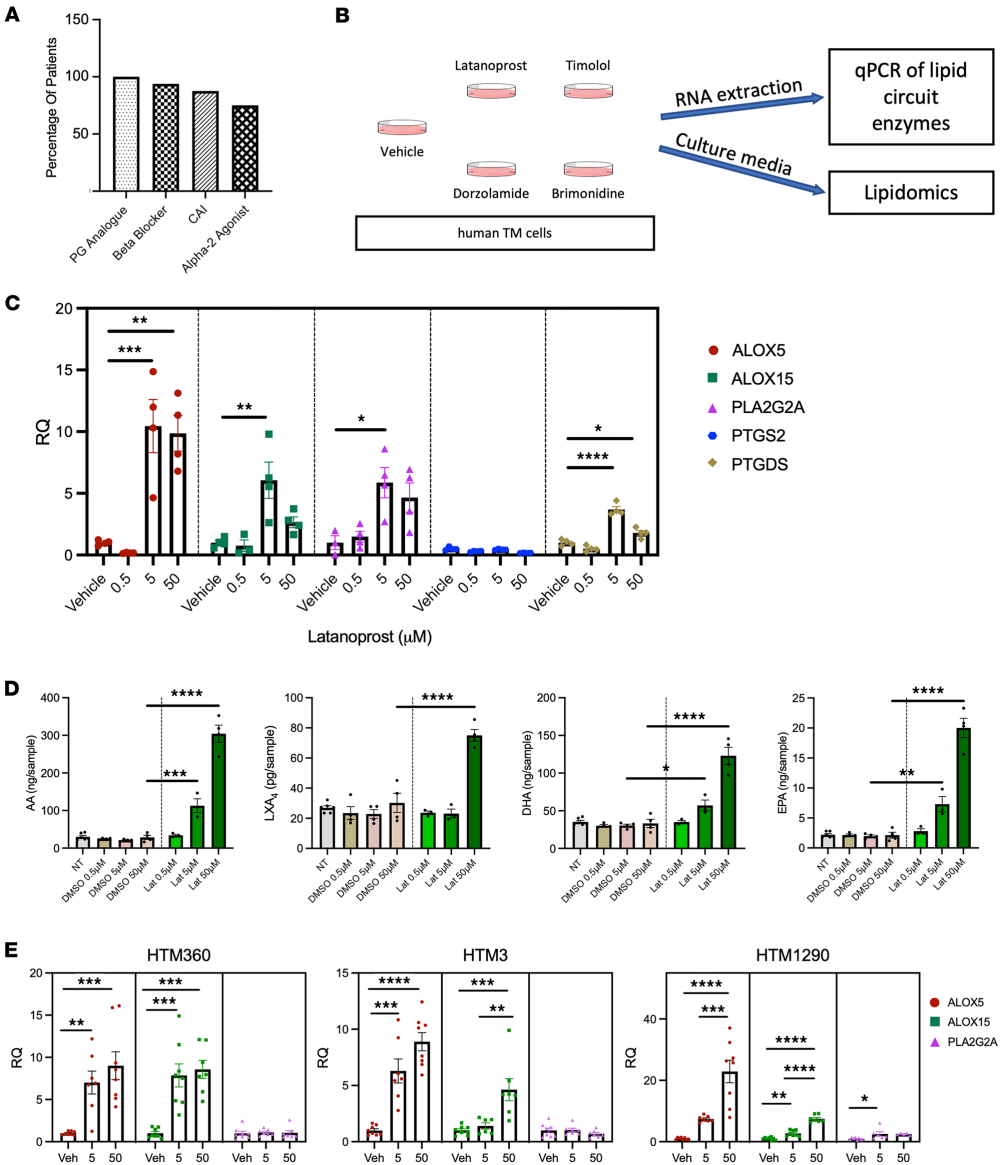
The AA/lipoxin circuit is induced by latanoprost and timolol treatment in human TM cells. (**A**) Graph representing the percentage of patients with glaucoma taking topical glaucoma eye drops, including prostaglandin (PG) analogs, beta blockers, carbonic anhydrase inhibitors, and α-2-adrenergic agonists. (**B**) A human TM cell line was treated with latanoprost (prostaglandin analog), timolol (beta blocker), dorzolamide (carbonic anhydrase inhibitor), brimonidine (α-2 adrenergic agonist), or vehicle for 1 hour before collecting RNA for qPCR and conditioned media for lipidomic analyses. (**C**) Quantification of qPCR results showed that treatment with latanoprost caused a significant, dose-dependent upregulation of *ALOX5*, *ALOX15*, *PLA2G2A*, and *PTGDS* expression, increasing from 0.5, 5, to 50 μM. (**D**) Lipidomic analyses of the culture media showed a significant increase in AA and LXA_4_ levels with increasing latanoprost treatment. In addition, EPA and DHA substrate levels were significantly elevated by treatment compared with vehicle. (**E**) Treatment with latanoprost was repeated on primary human TM cells derived from 3 donors (HTM360, HTM3, and HTM1290) at 5 and 50 μM. Analyses of qPCR results demonstrates significantly increased expression of *ALOX5* and *ALOX15*, but not *PLA2G2A* in all 3 lines. ****P < 0.0001; ***P < 0.001; **P < 0.01; *P < 0.05 by 1-way ANOVA with Tukey’s multiple-comparison test for each transcript or analyte. Data are presented as mean ± SEM. AA, arachidonic acid; ALOX5, arachidonate 5-lipoxygenase; ALOX15, arachidonate 15-lipoxygenase; DHA, docosahexaenoic acid; EPA, eicosapentaenoic acid; LXA_4_, lipoxin A_4_; PLA2G2A, phospholipase A_2_ group IIA; PTGDS, prostaglandin D_2_ synthase; PTGS2, prostaglandin-endoperoxide synthase 2 (COX-2); RQ, relative quantification.

We had predicted that lipoxin levels would be reduced in patients with glaucoma due to their well-documented proresolving activities, and from reports of other chronic diseases that exhibit this pattern (40–44). Therefore, the marked elevation of LXA_4_ we observed in glaucomatous aqueous fluid was unexpected. Upon consideration, we hypothesized that this response might be caused by the topical IOP-lowering medications the patients were taking. In particular, the most commonly prescribed glaucoma drug, latanoprost (6–8), is a prostaglandin F_2α_ (PGF_2α_) analog that could impact LOX or COX pathways and production of other AA metabolites. Notably, all enrolled patients with glaucoma were taking topical glaucoma medications, and usually more than one class, with 100% taking PGF_2α_ analogs, 93.75% on beta blockers, 87.5% on carbonic anhydrase inhibitors, and 75% taking α-2-adrenergic agonists (Figure 2A). Therefore, a majority of patients were typically prescribed combinations of these drug classes.

In order to test the potential influence of these medications on the AA/lipoxin circuit, an in vitro experiment was designed with relevant human cells. Based on our human outflow tissue staining for lipoxin synthetic enzymes (5-LOX and 15-LOX), a human TM cell line was directly treated with clinically relevant concentrations of common drugs representing each of the 4 administered classes, or vehicle, and analyzed for changes in eicosanoid and lipoxin pathways. After 1 hour of drug exposure, RNA was isolated from the treated cells to assess expression of relevant enzymes using reverse transcription quantitative polymerase chain reaction (RT-qPCR). In parallel, conditioned media were also collected for corresponding lipidomic analyses (Figure 2B).

Interestingly, latanoprost treatment significantly induced dose-dependent expression of key enzymes for generating lipoxins. Expression of the rate-limiting enzyme, 5-LOX (*ALOX5*), was markedly upregulated by 10.45- and 9.86-fold (*P* = 0.001) in human TM when treated with 5 μM and 50 μM latanoprost, respectively, compared with vehicle treatment (Figure 2C). Similarly, the second required enzyme for lipoxin formation, 15-LOX (*ALOX15*), was upregulated 6.06-fold by 5 μM latanoprost (*P* = 0.003). An enzyme that generates AA from phospholipids, phospholipase A_2_ (PLA_2_, gene *PLA2G2A*) was also upregulated 14.23-fold by 5 μM latanoprost (*P* = 0.015). Latanoprost did not induce expression of COX-2 (*PTGS2*), but increased expression of PGD_2_ synthase (*PTGDS*), which was upregulated 3.68-fold (*P* < 0.0001) and 1.79-fold (*P* = 0.02) by 5 μM and 50 μM latanoprost treatment, respectively (Figure 2C).

In parallel, conditioned media samples from this experiment were also analyzed by lipidomics for functional changes in lipoxin pathways. Consistent with the increases observed in *PLA2G2A*, *ALOX5*, and *ALOX15* gene expression, corresponding AA and LXA_4_ levels were significantly elevated in latanoprost-treated samples in a dose-dependent manner (Figure 2D). Compared with vehicle, 5 and 50 μM latanoprost treatment caused 5.32- and 10.7-fold increased levels of AA (*P* = 0.0002 and *P* < 0.0001, respectively), and LXA_4_ levels were significantly increased (2.48-fold) with 50 μM latanoprost (*P* < 0.0001). Interestingly, levels of additional substrates that are released by PLA_2_ were also increased, including EPA (5 and 50 μM latanoprost; *P* = 0.002 and *P* < 0.0001, respectively), and DHA (5 and 50 μM latanoprost; *P* = 0.002 and *P* < 0.0001, respectively; Figure 2D).

Of the other drug classes tested, a mixed picture was presented. Some surprisingly similar trends to that of latanoprost were observed in cells treated with the β-adrenergic receptor antagonist timolol; *ALOX5* was upregulated 8.12-fold (*P* < 0.0001) by 100 μM timolol treatment compared with vehicle. *ALOX15* was upregulated 3.22-, 3.23-, and 5.98-fold by 1, 10, and 100 μM timolol, respectively (*P* = 0.03, 0.03, and <0.0001; Supplemental Figure 4A). *PLA2G2A* was upregulated 16.34-, 11.82-, and 11.79-fold by 1, 10, and 100 μM timolol, respectively (*P* = 0.0002, 0.003, and 0.003, respectively), and *PTGDS* was upregulated 4.49-, 5.29-, and 10.28-fold by 1, 10, and 100 μM timolol, respectively (*P* = 0.007, 0.002, and <0.0001, respectively; Supplemental Figure 4A). In contrast, neither treatment with the carbonic anhydrase inhibitor dorzolamide nor α-2-adrenergic agonist brimonidine showed substantial effects on expression of the same gene panel (Supplemental Figure 5, A and B). Consistent with the RT-qPCR results, treatment with 10 and 100 μM timolol significantly elevated the levels of AA (16.46- and 19.37-fold, respectively; *P* < 0.0001 for both), and 100 μM timolol elevated levels of LXA_4_ (1.74-fold, *P* = 0.03; Supplemental Figure 4B). Concentrations were also elevated at 10 and 100 μM for EPA (*P* < 0.0001) and DHA (*P* < 0.0001; Supplemental Figure 4B). However, neither dorzolamide nor brimonidine generated substantial or consistent changes in AA, EPA, DHA, or LXA_4_ levels (Supplemental Figure 5, C and D).

Finally, to confirm the prominent latanoprost findings, our key RT-qPCR results were repeated in 3 distinct primary donor-derived human TM (HTM) cell populations, HTM360, HTM1290, and HTM3. Similar to the cell line results, expression of *ALOX5* and *ALOX15* was significantly elevated in a dose-dependent manner following latanoprost treatment at 5 and 50 μM (*P* < 0.001). However, *PLA2G2A* showed no significant increase, indicating a specific focus on the lipoxin synthetic route (Figure 2E). Together, these results present a picture that is fairly consistent with the patient lipidomic data, indicating that latanoprost, and to a lesser extent timolol, treatment specifically amplifies the AA/lipoxin synthetic circuit in TM cells.

### The AA/lipoxin circuit is induced by latanoprost in vivo, but not ocular hypertension alone.

Based on our clinical results, an alternative possibility is that ocular hypertension (OHT) alone induces the AA/LXA_4_ pathway. Yet, it was not possible to acquire untreated clinical aqueous humor samples from patients with glaucoma due to ethical considerations. To date, published transcriptomic data from OHT and glaucoma studies in human TM cells report no differential expression of the synthetic *ALOX5* or *ALOX15* genes due to elevated IOP or simulated OHT (45–48). Furthermore, we mined data from a published RNA-seq study that analyzed human TM cells with a gene knockdown genetically linked to POAG. Although a full differential analyses was not possible on these limited datasets, the results confirmed the expression of LXA_4_ synthetic genes in HTM cells, and suggest no substantive changes (Supplemental Figure 6).

**Figure 3:**
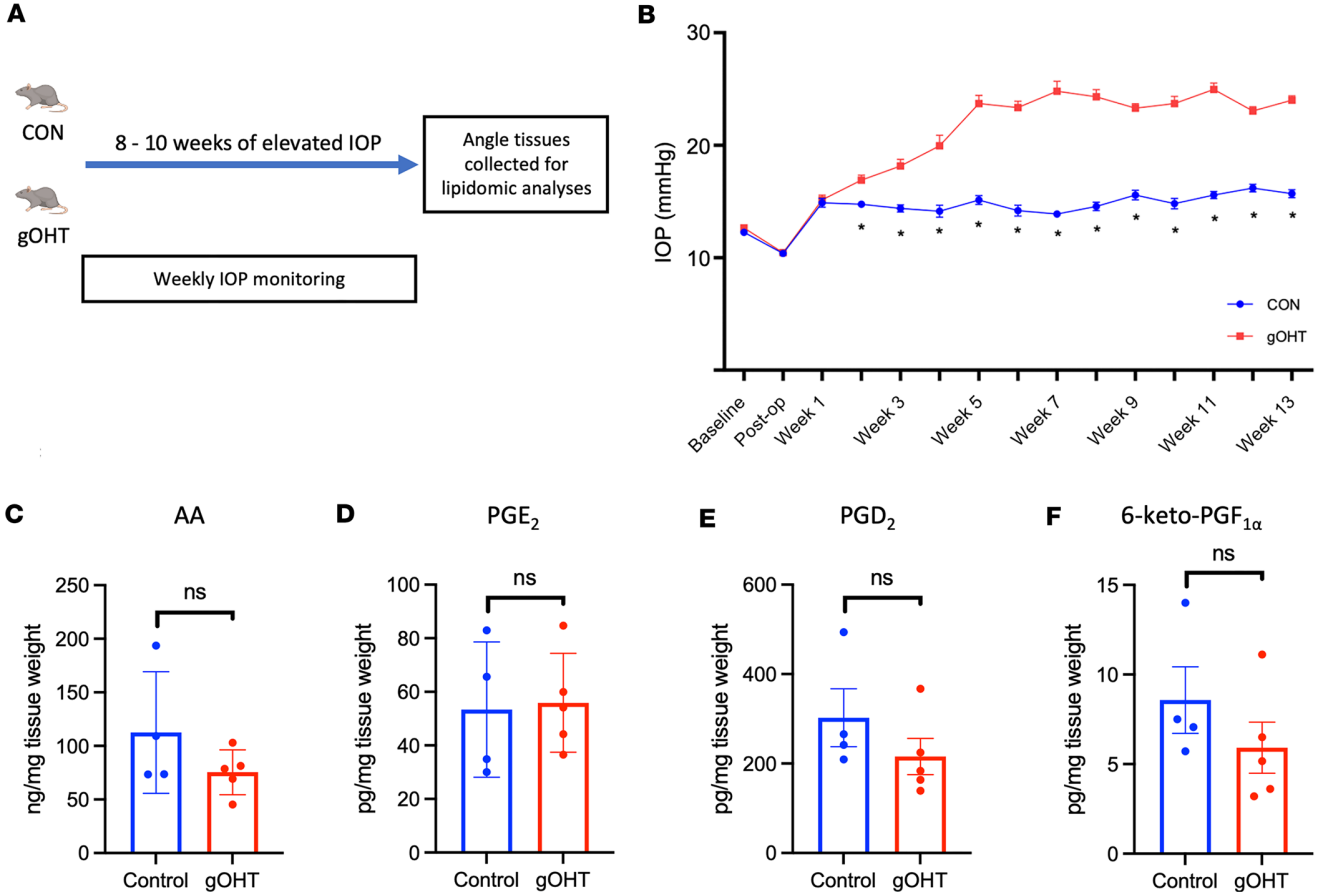
The AA/lipoxin circuit is not induced by OHT alone. (**A**) Gradual ocular hypertension (gOHT) was induced by a circumlimbal suture in 6-week-old Long Evans rats and maintained for 8–10 weeks before eyes were collected for lipidomic analyses. (**B**) As expected, circumlimbal suturing induced a gradual increase in IOP, consistently exceeding 20 mmHg from weeks 3 to 5 after suturing. *P < 0.0001 by 2-tailed, unpaired *t* test at each time point. Data are presented as mean ± SEM. (**C**) Lipidomic analyses detected AA levels that were not significantly altered in the OHT group compared to control. (**D**–**F**) Similarly, endogenous PGD_2_, PGE_2_, and 6-keto PGF_1α_ were detected, but were not significantly altered by OHT alone. NS, not significant by 2-tailed, unpaired *t* test. Data are presented as mean ± SEM. PGD_2_, prostaglandin D_2_; PGE_2_, prostaglandin E_2_.

In order to test the possibility of IOP-induced LXA_4_ production experimentally, we turned to a recently characterized rat model of gradual OHT (gOHT) generated by slack circumlimbal sutures that tighten over time (49). This model provides a consistent, inducible method to gradually elevate IOP without an underlying genetic mutation, dysmorphism, or introduction of foreign material into the outflow tissues. Once elevated, OHT was maintained for 8 weeks in the sutured eyes, at which time they were processed for lipidomic analyses of angle tissues (Figure 3, A and B). In these OHT samples, AA concentrations were not significantly different from control normotensive eyes, and the trend was toward reduced levels (Figure 3C). Likewise, levels of PGE_2_, PGD_2_, and 6-keto PGF_1α_ were detected, but were not significantly altered and exhibited a similarly reduced trend (Figure 3, D–F). Notably, LXA_4_ levels analyzed from each single eye were below the detection threshold for all groups, which is likely due to the small tissue sample sizes. Together, these findings contrast with our clinical lipidomic results and indicate that OHT by itself likely does not increase LXA_4_ formation.

**Figure 4:**
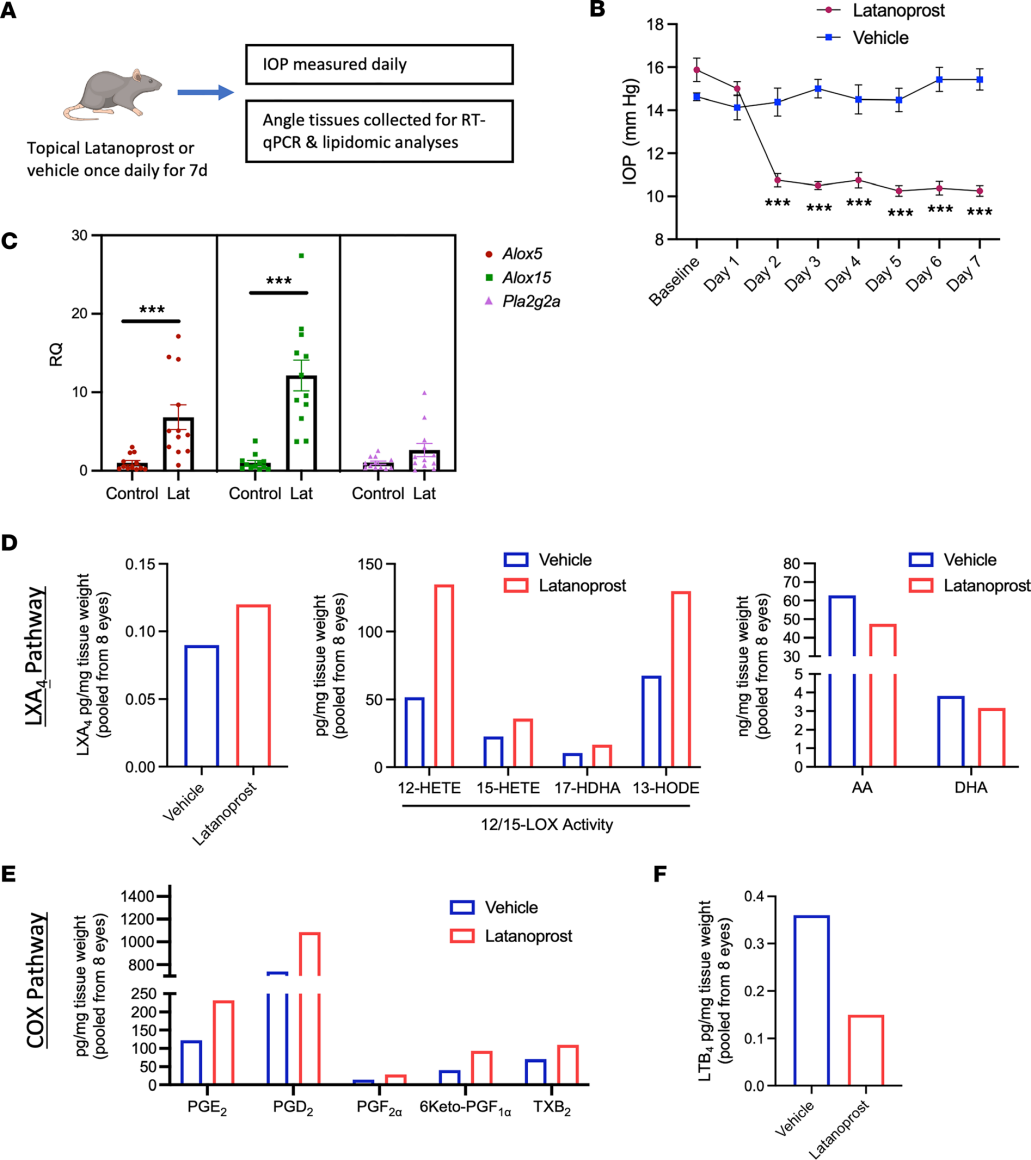
The LXA_4_ and COX pathways are induced by latanoprost treatment in vivo. (**A**) Six-week-old Long Evans rats were administered topical latanoprost for 7 days, followed by the analyses of angle tissues. (**B**) Latanoprost treatment resulted in sustained IOP reduction over the treatment period. ***P < 0.001 by 2-tailed, unpaired *t* test at each time point. Data are presented as mean ± SEM. (**C**) Expression of *Alox5* and *Alox15* were significantly increased in angle tissues, but not *Pla2g2a*. ***P < 0.0001 by 2-tailed, unpaired *t* test for each transcript. Data are presented as mean ± SEM. (**D**) Lipidomic analyses detected increased levels of LXA_4_ in latanoprost-treated samples compared with vehicle, along with several pathway intermediates and products, including 12-HETE, 15-HETE, 17-HDHA, and 13-HODE. Concentrations of AA and DHA were slightly reduced (*n* = pooled samples from 8 eyes). (**E**) Elevated COX pathway products were also detected, including PGE_2_, PGD_2_, PGF_2α_, 6-keto-PGF_1α_, and Thromboxane B_2_ (TXB_2_). (*n* = pooled samples from 8 eyes). (**F**) Levels of LTB_4_ were sharply reduced in latanoprost-treated samples compared with vehicle (*n* = pooled samples from 8 eyes).

In comparison with OHT, topical administration of latanoprost or vehicle to rat eyes alone resulted in lipid mediator profiles that largely overlapped with the clinical and human cell culture results. Rats were dosed daily with 40 μL of latanoprost (0.005%) or vehicle for 7 days, and the angle tissues collected for RT-qPCR and lipidomic analyses (Figure 4, A and B). Consistent with the previous in vitro data, expression of both *Alox5* and *Alox15* was significantly elevated (*P* < 0.0001), while *Pla2g2a* showed an increasing trend that did not reach significance (Figure 4C). For lipidomic analyses, this time the sample homogenates were pooled (*n* = 8) to improve detection of intermediates and LOX products in these small rat tissue samples. Levels of LXA_4_ were correspondingly elevated in latanoprost-treated samples compared with vehicle controls, along with several key pathway intermediates and products of the lipoxin biosynthetic pathway, similar to the clinical and human TM cell samples (Figure 4D). In comparison, the PUFA substrates AA and DHA were slightly reduced by latanoprost treatment (Figure 4D). Interestingly, a panel of intermediates and products of the COX pathway were also elevated, including PGE_2_ and PGD_2_, as in the clinical samples (Figure 4E). Finally, leukotriene B_4_ (LTB_4_), a 5-LOX product, was sharply reduced (Figure 4F), in sharp contrast to the increase in LXA_4_; this pattern is consistent with a shift in 5-LOX activity toward generating SPMs instead of proinflammatory leukotrienes (50, 51). In comparison, timolol was also administered topically with the same experimental design, and generally resulted in no substantial changes in LXA_4_ or COX pathway products in the rat model (Supplemental Figure 7). Taken together, these data provide direct evidence that latanoprost specifically promotes AA metabolism and LXA_4_ synthesis, as well as prostaglandin production in vivo.

### LXA_4_ does not cause acute IOP lowering but inhibits proinflammatory cytokines and induces production of TGF-β_3_.

**Figure 5:**
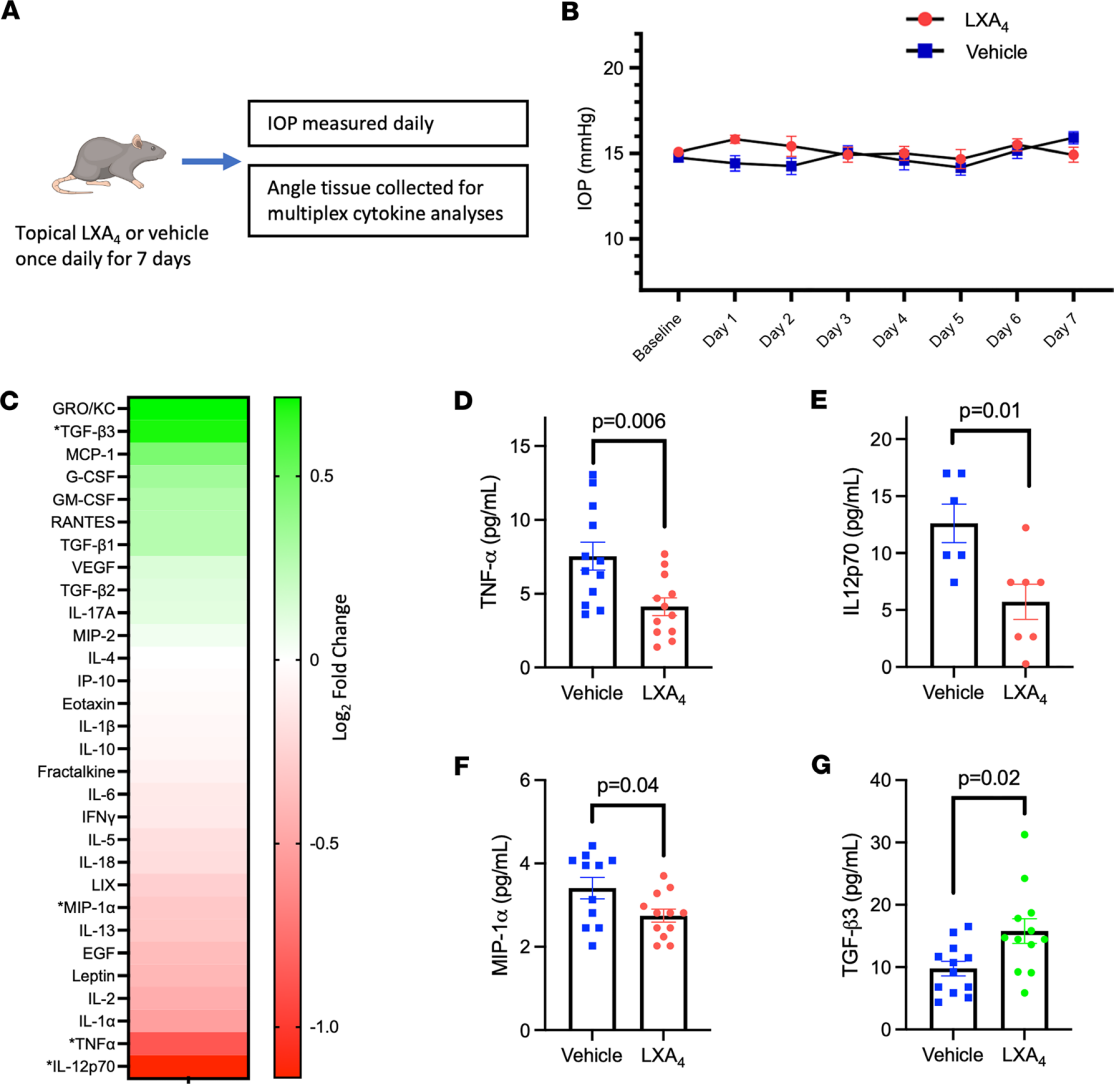
LXA_4_ does not cause acute IOP lowering, but inhibits proinflammatory cytokines and induces production of TGF-β. (**A**) Six-week-old Long Evans rats were treated with LXA_4_ once daily for 1 week, and the eyes were collected for cytokine analyses of the angle tissues. (**B**) Daily IOP measurements indicate that LXA_4_ did not cause significant IOP changes compared to vehicle treated controls (no significant differences by 2-tailed, unpaired *t* test for each time point; data are presented as mean ± SEM). (**C**) At the end of the study, angle tissue samples were subjected to a panel of 30 cytokines, presented here as a heatmap of log_2_(fold change) normalized to vehicle tissue levels (significantly altered cytokines are marked with an asterisk). (**D**–**G**) Individual data are presented for significantly altered cytokines, including significant decreases in the levels of (**D**) TNF-α, (**E**) IL-12, and (**F**) MIP-1α, and (**G**) a significant increase in TGF-β_3_ levels (*P* values indicated by 2-tailed, unpaired *t* test; data are presented as mean ± SEM). IL-12p70, interleukin-12p70; MIP-1α, macrophage inflammatory protein-1α; TGF-β_3_, transforming growth factor β_3_; TNF-α, tumor necrosis factor α.

To study the effect of elevated LXA_4_ itself on IOP and the outflow tissues, 6-week-old Long Evans rats were treated topically by eye drop with 40 μM LXA_4_, once daily, for 7 days (Figure 5A). IOP was monitored during the first 24 hours and then daily until the end of the experiment. Over this period there was no significant difference in IOPs between LXA_4_-treated and vehicle-treated eyes (Figure 5B). This indicates that LXA_4_ alone is not sufficient to reduce IOP.

However, as LXA_4_ has potent antiinflammatory activities (40, 52, 53), we also investigated whether repeated treatment would alter inflammation signaling in outflow tissues. Angle tissues were harvested from vehicle- and LXA_4_-treated rat eyes and subjected to a cytokine panel of 30 mediators (Figure 5C, full results in Supplemental Table 2). Significantly altered cytokines were interleukin-12 (IL-12; 12.60 vs. 5.73 pg/mL, *P* = 0.01), macrophage inflammatory protein-1α (MIP1α; 3.41 vs. 2.75 pg/mL, *P* = 0.04), and tumor necrosis factor α (TNF-α; 7.55 vs. 4.12 pg/mL, *P* = 0.006), whose levels were all significantly lower in LXA_4_-treated eyes (Figure 5, D–F). These results are consistent with expected antiinflammatory effects. In comparison, TGF-β_3_ concentrations were significantly higher in LXA_4_-treated eyes compared with vehicle-treated eyes (15.79 vs. 9.78 pg/mL, *P* = 0.02; Figure 5G). TGF-β_3_ is part of a superfamily of cytokines that promote ECM remodeling. We note that other prominent family members, TGF-β_2_ and TGF-β_1_, were also elevated in LXA_4_-treated samples, although these changes did not reach significance.

### Prostaglandin synthesis is required for latanoprost IOP-lowering activity.

**Figure 6:**
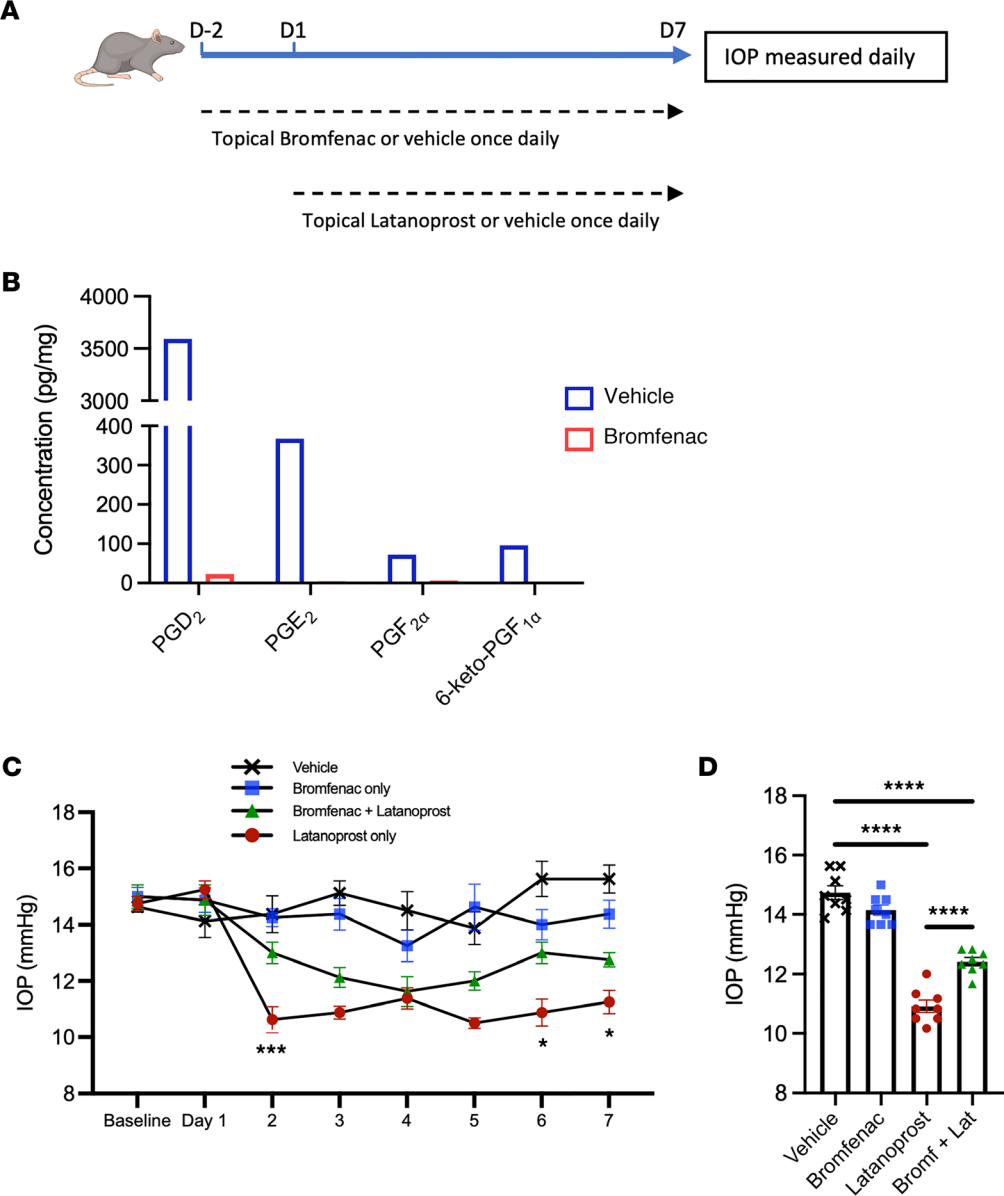
Prostaglandin synthesis is required for latanoprost acute IOP-lowering activity. (**A**) Six-week-old Long Evans rats were treated with the COX inhibitor, bromfenac, or vehicle for 48 hours prior to starting concomitant latanoprost treatment over the next 7 days. Both treatments were administered once daily, with daily IOP monitoring. (**B**) Analyses of rat angle tissues following administration of bromfenac or vehicle showed strong inhibition of levels of PGD_2_, PGE_2_, PGF_2α_, and 6-keto PGF_1α_ (bars are pooled composites of 6 samples). (**C**) Bromfenac treatment alone had no effect on IOP, while latanoprost treatment caused a rapid and sustained IOP reduction. When administered together, bromfenac treatment attenuated the IOP-lowering activity of latanoprost. ***P < 0.001, *P < 0.05 between latanoprost and latanoprost + bromfenac by 1-way ANOVA with Tukey’s multiple-comparison test at each time point. Data are presented as mean ± SEM. (**D**) Comparison of the average IOP from days 2 to 7 demonstrated significant attenuation of the IOP-lowering effect of latanoprost by bromfenac. ****P < 0.0001 by 1-way ANOVA with Tukey’s multiple-comparison test. Data are presented as mean ± SEM. COX, cyclooxygenase.

As LXA_4_ did not mediate acute IOP lowering, we wondered whether this component of latanoprost activity might be generated by another branch of AA metabolism to generate an autocrine cycle of prostaglandin synthesis. Therefore, we sought to block prostaglandin production in the context of latanoprost treatment. The 2 COX enzymes, COX-1 and COX-2, catalyze the formation of prostaglandins from AA (54). Bromfenac preferentially inhibits COX-2, although it also targets COX-1, and demonstrates potent antiinflammatory effects by blocking prostaglandin synthesis (55). Bromfenac is widely used in the eye after cataract surgery to decrease the risk of cystoid macular edema secondary to ocular inflammation (56). Rat eyes were treated with topical bromfenac daily for 2 days before initiating daily latanoprost eye drops for 1 week. In select groups, bromfenac administration was continued during the 7 days of latanoprost treatment (Figure 6A). Lipidomic analyses of angle tissues showed strong inhibition of prostaglandin synthesis by bromfenac (Figure 6B). Upon IOP measurement, eyes treated with bromfenac alone showed no IOP change compared to vehicle. As expected, treatment with latanoprost alone significantly lowered IOP. However, eyes treated with both bromfenac and latanoprost exhibited a significantly reduced activity compared with latanoprost alone (Figure 6C). Quantification of average IOP between days 2 and 7 (when latanoprost showed maximal IOP lowering) revealed a significant difference between latanoprost treatment alone, and cotreatment with bromfenac and latanoprost (*P* < 0.001, Figure 6D). These findings indicate that synthesis of endogenous prostaglandins is required for full IOP-lowering actions of latanoprost.

## Discussion

**Figure 7:**
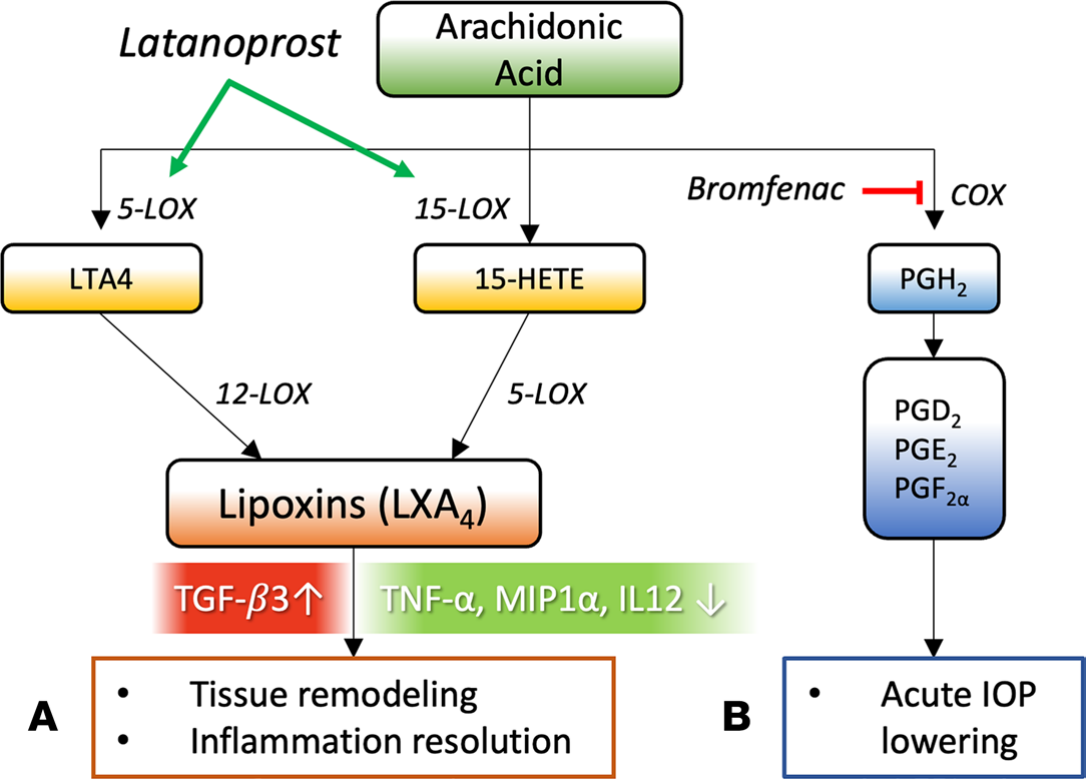
Flowchart depicting proposed parallel AA-dependent drug mechanisms. (**A**) The upregulation of 5-LOX and 15-LOX by latanoprost induces synthesis of LXA_4_, resulting in tissue remodeling and inflammation resolution to exert sustained IOP-lowering effects. (**B**) In concert, endogenous prostaglandins are generated via COX activity, resulting in acute IOP lowering. PGD_2_, prostaglandin D_2_; PGE_2_, prostaglandin E_2_; PGF_2α_, prostaglandin F_2α_.

The production and roles of lipid mediators derived from PUFAs through the LOX and COX pathways remain surprisingly unexplored in patients with glaucoma. We report that aqueous humor lipidomic analyses of COX- and LOX-derived mediators showed a strong and selective upregulation of the AA/LXA_4_ pathway in patients with glaucoma compared with non-glaucomatous controls. This was a strikingly selective result, considering the panel of 40 mediators and intermediates assessed, including the ω-3 substrates DHA and EPA, along with a variety of active metabolites. This robust increase in LXA_4_ was surprising, given its established protective and antiinflammatory actions. Yet, LXA_4_ formation can be promoted pharmacologically in other contexts (33–38). Since all patients with glaucoma included in this study were unavoidably taking topical IOP-lowering medications, including prostaglandin mimetics, we evaluated their direct effect on the AA/LXA_4_ pathway. Together, our results indicate that latanoprost induces a specific and dose-dependent increase in LXA_4_ production in vitro and in vivo. This result is supported by an early study in human blood leukocytes that reported the ability of another prostaglandin, PGE_2_, to induce lipoxin formation de novo by regulating 15-LOX expression (50). In contrast, OHT or TM cell dysfunction alone did not appear to activate this pathway in vitro or in vivo. In turn, exogenous LXA_4_ had no acute effect on IOP, but strongly inhibited proinflammatory cytokines and stimulated production of TGF-β_3_. In a parallel pathway, COX-mediated prostaglandin synthesis was required for the acute IOP-lowering effects of latanoprost. Together, these results suggest a new model for parallel acute and long-term latanoprost mechanisms that can be uncoupled to involve either prostaglandin or lipoxin actions, respectively (Figure 7).

PGAs are widely used as the first-line treatment for POAG due to their once-daily dosing regimen and substantial IOP reduction (6–8, 57). Latanoprost alone is one of the most commonly prescribed medications, with nearly 10 million prescriptions in the United States in 2021 (https://clincalc.com/DrugStats/Drugs/Latanoprost). Yet, as a class, the mechanisms underlying these drug actions are still unclear. Latanoprost is an analog of PGF_2α_, with an isopropyl ester substituent replacing the α-carboxylic acid. It is thought to lower IOP by increasing the outflow of aqueous humor through the uveoscleral (10) and TM pathways (58, 59). The canonical mechanism of action of latanoprost involves binding to a G protein–coupled FP receptor, which is expressed in the ciliary muscle and TM of the eye (12). Traditionally, activation of the FP receptor by PGF_2α_ analogs is thought to stimulate PLA_2_, resulting in the release of AA and subsequent synthesis of endogenous prostaglandins, including PGE_2_. Production of PGE_2_ induces cAMP that promotes smooth muscle relaxation to enhance aqueous humor outflow and reduce IOP. Short-term treatment in primates with PGF_2α_ results in rapid IOP reduction, and normalization after cessation of treatment (60–62). We observed some increased prostaglandin synthesis following latanoprost treatment. Also, inhibition of endogenous prostaglandin production by blocking COX activity significantly reduced the acute IOP-lowering actions of latanoprost. These results are consistent with a pseudo-autocrine loop contributing to acute IOP lowering, as previously proposed (63). Conflicting results have been reported for the effects of topical nonsteroidal antiinflammatory drugs in patients with glaucoma using PGAs (64, 65), and our results suggest more research in this area is needed.

In addition to mediating transient IOP lowering, increasing evidence describes how PGAs also remodel the ECM of the TM and ciliary body via TGF-βs and MMPs (16, 66–71). In contrast with topical PGAs (which typically require uninterrupted daily dosing and are rarely withdrawn in routine practice), intracameral sustained-release prostaglandin implants elute drug for approximately 3–4 months, after which IOP is observed in the absence of further treatment. In a subset of patients, clinically meaningful IOP reduction persists for up to 18–24 months beyond the elution period (17, 72), and decreased anterior scleral thickness following PGA treatment has been linked to a similar mechanism (73). The biological basis for this response remains unclear. One proposed explanation is that transient prostanoid exposure may induce longer-lasting structural or ECM changes within the outflow pathway (13, 15). Yet, the mechanism that directs this tissue remodeling pathway is not well understood (15). Our results indicate that 5-LOX and 15-LOX are localized to human outflow tissue, and that treatment with latanoprost directly upregulates their expression in TM cells, resulting in increased synthesis of LXA_4_. Supplementation of LXA_4_ results in a marked antiinflammatory effect, strongly inhibiting TNF-α, IL-12p70, and MIP1. We also observed increased production of TGF-β_3_, which has been directly linked to TM remodeling (66, 74, 75), and trends toward increased TGF-β_1_ and TGF-β_2_. Thus, we have identified what we believe is a novel branch of latanoprost signaling that provides a biochemical connection to ECM remodeling and inflammation inhibition via LXA_4_ production (Figure 7). Our analyses of published transcriptomic data establish that human TM cells express the LXA_4_ receptor ALX/FPR2 (46). It will be important to further clarify the LXA_4_-induced signaling mechanism in future studies.

Interestingly, similar to latanoprost, timolol also induced upregulation of PLA_2_, 5-LOX, and 15-LOX in vitro, with increased synthesis of AA and LXA_4_. β-Adrenergic antagonists act primarily through decreased cytosolic cAMP levels and altered calcium signaling (76). Activation of the AA/LXA_4_ pathway requires calcium signaling, which may partially explain these unexpected actions. However, these timolol results were not repeated in rat eyes in vivo. Timolol canonically reduces IOP by decreasing aqueous humor secretion (77), but recently has also been reported to alter aqueous outflow facility in healthy human eyes through an unknown mechanism (78). Upregulation of AA and its downstream mediators by timolol suggests a potential common or interacting mechanism of action with latanoprost. Yet, the detailed interactions that mediate these effects will require further clarification.

In summary, although prostaglandin analog use is widespread, the molecular mechanisms underlying their actions in the eye are still unclear. We report an upregulation of AA/LXA_4_ induced by latanoprost that may explain long-term effects. Given the well-established proinflammatory roles of PGF_2α_ (79), activation of this unanticipated pathway results in antiinflammatory and remodeling changes in the outflow tissues that can be uncoupled from its acute IOP-lowering effects. To the best of our knowledge, the roles of AA and its LOX products have not been explored before in the context of glaucoma and IOP-lowering treatments. Therefore, these insights may provide a foundation for investigating new therapeutic targets for OHT with sustained antiinflammatory and remodeling actions, and for comparing with corresponding proteomic and transcriptomic datasets (80–82). Finally, our findings also suggest a note of caution, as clinical samples are accompanied by a variety of environmental variables that cannot be well controlled. In particular, similar patient biomarker studies should be carefully interpreted to distinguish observations due to the disease process from changes resulting from treatments.

## Methods

### Sex as a biological variable.

Our study examined matched male and female clinical samples, and similar findings are reported for both sexes (Table 1). For rat experiments, as no sex-dependent differences had been detected in clinical samples, or during previous and pilot rat model studies, only male rats were used for consistency.

### Patient recruitment and sample collection.

Patients with a diagnosis of POAG, aged 60–80 years, and scheduled for glaucoma surgery with or without cataract surgery at Toronto Western Hospital or Kensington Eye Institute were approached for inclusion in the study. Age-matched control samples were obtained from patients without glaucoma undergoing routine cataract surgery. Patients with diabetes mellitus, systemic inflammatory disease, uveitis, retinopathy, and age-related macular degeneration, or those taking nonsteroidal antiinflammatory drugs were excluded. All enrolled patients with glaucoma were taking topical glaucoma medications, and usually more than one class, with 100% taking PGF_2α_ analogs, 93.75% on beta blockers, 87.5% on carbonic anhydrase inhibitors, and 75% taking α-2-adrenergic agonists. From each eye, 100 μL of aqueous humor was collected using a 30-gauge needle mounted on a 1-mL syringe, introduced into the anterior chamber anterior at the limbus, prior to any surgical intraocular entry. The samples were immediately snap frozen on dry ice and stored at –80°C until assessment by lipidomic analyses.

### Lipidomic analyses.

Tissues were processed by placing them in tubes containing sterile ceramic beads and methanol (MeOH). Tissues were completely homogenized in a refrigerated bead homogenizer and MeOH supernatants were collected by high-speed centrifugation at 4°C. For lipidomic analyses, aqueous humor, cell media, or supernatants from homogenized tissue samples were extracted using solid-phase C-18 columns. Samples were collected from the column by LC/MS grade MeOH and the solvent evaporated under a gentle stream of nitrogen. Samples were immediately resuspended in 50:50 MeOH/water (LC/MS grade) and loaded into a refrigerated autosampler for LC-MS/MS analysis. Four hundred picograms of class-specific deuterated (-d) internal standards (AA-d8, DHA-d5, PGE_2_-d4, lipoxin A_4_-d5, LTB_4_-d4, and 15-hydroxyeicosatetraenoic acid-d8) were added to each sample prior to processing and extraction to calculate the recovery of specific classes of PUFA and LOX and COX pathway metabolites. Lipid mediators were identified and quantified using an Applied Biosystems SCIEX QTRAP 4500 mass spectrometer system consisting of an Agilent 1200 Series high-pressure LC system with a refrigerated 96-well autosampler and Phenomenex Luna C18 column (150 × 2 mm, 5 μM, 100 Å). Mobile phases used were A (71.9% water/28% acetonitrile/0.1% acetic acid [vol/vol/vol]) and B (60% isopropanol/40% acetonitrile [vol/vol]) that were run as a gradient. The analyses included PUFAs (AA, DHA, and EPA), their downstream mediators (prostaglandins, leukotrienes, lipoxins, resolvins, and maresins), and their metabolic precursors (monohydroxy-PUFAs) and metabolites, as previously published (28, 83–85). Note: These analytes are structurally and functionally distinct from membrane phospholipids, assessed in a single previous study of glaucomatous aqueous humor (86). PUFAs and their metabolites were analyzed by scheduled multiple reaction monitoring (MRM), a sensitive method for targeted analysis in ABSCIEX triple quadrupole instruments that isolates a specific precursor ion, fragments the ion, and monitors structure-specific fragment ions. MRM was used in the negative ion mode using 3 to 4 structure-specific fragment transition ions and matching retention times with authentic internal and external standards as the criteria for identification. A signal-to-noise ratio above 5:1 for the diagnostic ion in the raw MS chromatogram was set as the threshold for quantification. The mass/charge ratio (*m/z*) of precursor/diagnostic quantifying fragment ions and LC retention times for analytes and internal deuterated standards were recently published (85). Quantification, calibration curves, and HPLC retention times for each analyte were established with authentic synthetic standards (Cayman Chemical) (Supplemental Figures 1 and 2).

### Human TM cell culture.

For preliminary experiments, human TM-1 cells (in-house) were cultured in low-glucose Dulbecco’s modified Eagle medium (DMEM; Sigma-Aldrich, D-5523), 10% premium fetal bovine serum, 2 mM L-glutamine, 50 μg/mL gentamicin sulfate, and Primocin (Invivogen, ant-pm-1) (87). Cells were grown in 10-cm plates at 8% CO_2_ and media were changed every 2 days. Upon reaching confluence, cells were treated with latanoprost (CAS no. 130209-82-4, MilliporeSigma), timolol maleate (CAS no. 26921-17-5, MilliporeSigma), dorzolamide (CAS no. 120279-96-1, MilliporeSigma), or brimonidine (CAS no. 59803-98-4, MilliporeSigma) for 1 hour at the indicated concentration. Based on their established activities, each drug was dissolved in DMSO to the following concentrations: latanoprost, 0.5, 5, and 50 μM; timolol, 1, 10, and 100 μM; dorzolamide 0.5, 5, and 50 μM; and brimonidine 2, 20, and 200 μM. Following treatment, cells were collected after 1 hour for RT-qPCR and the cell culture media were collected and snap frozen for lipidomic analyses. To confirm key latanoprost results, 3 primary-derived human TM cell populations were tested: HTM360, HTM1290 (both in-house), and HTM3 (a gift from J. West-Mays, Department of Pathology and Molecular Medicine, McMaster University, Hamilton, Ontario, Canada). HTM cells were isolated from healthy human donor eyes in accordance with the Declaration of Helsinki (88, 89) and characterized to be HTM cells based on previously described criteria (90). Briefly, cells were cultured in low-glucose DMEM (Sigma-Aldrich), supplemented with 2 mM L-glutamine, 2.5 μg/mL amphotericin B, 50 μg/mL gentamicin sulfate, and 1 ng/mL FGF-2. Following latanoprost treatment at 5 and 50 μM, RNA was collected and amplified by qPCR primers as indicated.

### RT-qPCR.

RNA was extracted from TM-1 cells using the RNeasy Mini Kit (Qiagen, 74104) according to the manufacturer’s instructions. RNA samples were treated with RNase-free DNase (Promega RQ1 kit, PR-M6101). RNA purity was assessed using a Nanodrop 2000 spectrophotometer (Thermo Fisher Scientific, ND-2000), followed by cDNA synthesis using the SuperScript IV First-Strand Synthesis system (Invitrogen, 18091050). RT-qPCR was performed using SYBR Green PCR Master Mix (Applied Biosystems, Thermo Fisher Scientific, 4309155) on an Eppendorf Realplex 2 Mastercycler. The genes and primers used are listed in Table 2. Amplification of mRNA was normalized to *GAPDH* and the 2^–ΔΔCt^ comparative quantification method was used (91).

### Animal experiments.

For all rodent experiments, 6-week-old Long Evans rats (Charles River Laboratories) were used. The gOHT model was performed as previously reported (49). Briefly, chronic OHT was induced using a nylon 8-0 circumlimbal suture on a tapered needle (8-0 sterile microsuture, AROSurgical Instruments) passed subconjunctivally 1.5 mm posterior to the limbus under intraperitoneal ketamine-xylazine anesthesia. After making 5–6 sequential subconjunctival passes all around the limbus, the suture was tied off using a slip knot anchored by 3 simple knots. The suture was left snug, taking care not to directly induce elevated IOP secondary to a tight suture. The sutures then were allowed to slowly tighten over time, resulting in gradual elevation of IOP. In all experiments, both eyes of each animal were subjected to the same treatment to avoid potential confounding contralateral effects.

### IOP measurement.

A Tonolab rebound tonometer (Icare) was used to measure the IOP according to the manufacturer’s directions. For each measurement, the tonometer tip was aligned perpendicular to the central cornea. Measurements were obtained at baseline prior to suturing or treatments, following 1 week of prior alternate-day measurements to familiarize the animal with the procedure. Measurements were obtained while the animal was awake between 11 am and 1 pm. Care was taken not to stress the animal or exert pressure on the periocular region during the IOP recordings. Each measurement with the Tonolab rebound tonometer itself consists of 6 separate readings, of which the highest and lowest are automatically excluded and the mean of the 4 middle readings are displayed as the final result by the device. For each animal and IOP monitoring session, the mean of 2 consecutive measurements was recorded if they were within 2 mmHg of each other; if there was more than a 2-mmHg difference, then the median of 3 measurements was recorded.

### Pathological analyses and staining.

After euthanasia, eyes were fixed in 4% paraformaldehyde, equilibrated in 30% sucrose, embedded in optimal cutting temperature compound, and cryosectioned. Sections (12 μm) were blocked with 5% donkey serum and probed with primary antibodies against 5-LOX (Novus Biologicals, catalog NB110-58748) and 15-LOX (Santa Cruz Biotechnology, catalog sc-133085) according to standard protocols. The sections were washed with PBS/Tween and incubated with fluorescently conjugated secondary antibodies (Molecular Probes) and DAPI. Subsequently, sections were mounted using MOWIOL 4-88 (MilliporeSigma). Immunofluorescence images were acquired with a Nikon Eclipse-Ti confocal microscope and analyzed with NIS Elements software version 4.51.

### Angle tissue dissection and homogenization.

Dissection of a 1-mm strip of rat angle tissue containing a small rim of overlying sclera and cornea, TM, peripheral iris, and ciliary body with ciliary processes was carefully performed using Vannas scissors and atraumatic fine forceps. The collected tissue sample was homogenized in aliquoted microfuge tubes, and then snap frozen at −80°C. Samples were then submitted to quantitative multiplex laser bead analyses (Bio-Plex 200) for assessment of a 27-plex rat cytokine panel and a 3-plex TGF-β panel (Eve Technologies) or lipidomic analysis.

### Statistics.

For all experiments, *n* refers to the number of eyes or biological replicates. GraphPad Prism 8.4.3 was used to generate graphs. IOP trend comparisons and lipidomic profile comparisons between 2 groups were performed using the unpaired *t* test. Comparisons between more than 2 groups were performed using 1-way ANOVA with Tukey’s post hoc analyses. A *P* value of less than 0.05 was considered statistically significant.

### Study approvals.

All human participants signed an informed consent form. This study was performed according to a protocol approved by the Research and Ethics Boards of University Health Network and Kensington Eye Institute, and adhered to the tenets of the Declaration of Helsinki. All animal procedures and protocols conformed to the guidelines of the ARVO statement for the use of animals in ophthalmic and vision research, and were approved by the University Health Network Animal Care Committee. All procedures were performed in accordance with all relevant regulations and are reported in accordance with ARRIVE guidelines.

### Data availability.

All study data are available in the published manuscript, accompanying supplemental material, or from the corresponding authors upon reasonable request. Values for all data points in graphs are reported in the Supporting Data Values file.

## Author contributions

DJM, JMS, and KG designed the study. DJM, YMB, MS, and GT collected patient samples and data, DJM, ILB, DC, and JWZ performed experiments and analyzed data. SM and JH performed lipidomic analyses. DMP provided materials and advised on study design, DJM, KG, and JMS wrote the manuscript. JGF, KG, and JMS obtained funding. All authors reviewed and edited the manuscript.

## Funding support

This work is the result of NIH funding, in whole or in part, and is subject to the NIH Public Access Policy. Through acceptance of this federal funding, the NIH has been given a right to make the work publicly available in PubMed Central.

- Canadian Institutes of Health Research grants PJT166201 and PJT168845 (to JS).
- NIH grant R01EY030218 (to JMS, JGF, and KG).
- NIH grants R01EY032905 and R01EY017006 (to DMP).

## Supplementary Material

Supplemental data

Supporting data values

## Figures and Tables

**Table 1:**
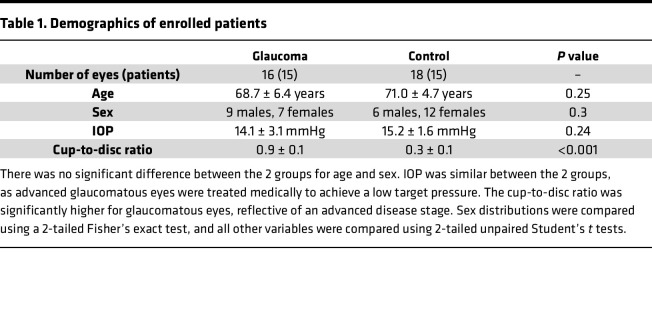
Demographics of enrolled patients

**Table 2:**
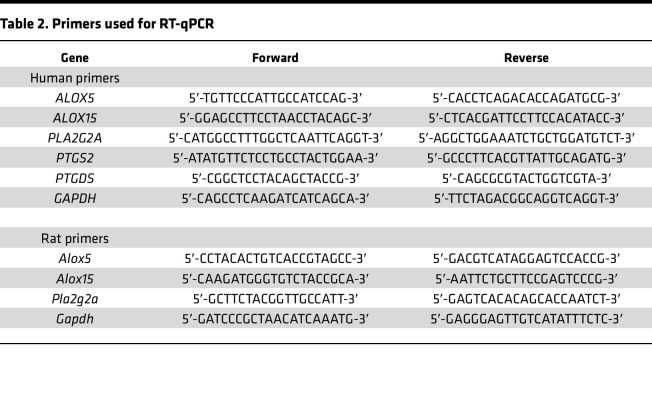
Primers used for RT-qPCR

## Version 1 02/24/2026

In-Press Preview

## Version 2 04/08/2026

Electronic publication
