# Supplementary material for: Lipidomic analysis reveals drug-induced lipoxin synthesis in glaucoma treatment
Source: JCI Insight. 2026 Feb 24;11(7):e192010. doi: 10.1172/jci.insight.192010 (PMC13134715; doi:10.1172/jci.insight.192010)
Supplement: Supplemental data [file jciinsight-11-192010-s038.pdf]

Supplementary Table 1: Aqueous humor lipidomic analysis results (values in pg/100 µL, *indicates a significant difference between glaucoma and control groups).

Analyte	Glaucoma	Control
**AA***	**1328.04 ± 312.43**	**643.07 ± 127.15**
DHA	212.03 ± 185.33	131.09 ± 21.76
EPA	6.21 ± 5.59	3.76 ± 0.31
5-HETE	ND	ND
12-HETE	ND	ND
15-HETE	ND	ND
20-HETE	ND	ND
5-oxo-ETE	ND	ND
4-HDHA	ND	ND
7-HDHA	ND	ND
14-HDHA	ND	ND
17-HDHA	ND	ND
**12-HEPE***	**1.38 ± 0.62**	**ND**
15-HEPE	ND	ND
18-HEPE	ND	ND
**13-HODE***	**12.17 ± 3.71**	**16.32 ± 1.27**
PGE_2	9.05 ± 12.28	ND
PGD_2	12.98 ± 7.77	ND
PGF_{2a}	ND	ND
6-keto-PGF_{1a}	ND	ND

Analyte	Glaucoma	Control
15-deoxy PGJ_2	ND	ND
**LXA_4***	**1.05 ± 0.36**	**0.74 ± 0.08**
LXB_4	ND	ND
LTB_4	ND	ND
6-trans-LTB_4	ND	ND
20-hydroxy-LTB_4	ND	ND
20-carboxy LTB_4	ND	ND
LTB_6	ND	ND
LTC_4	ND	ND
LTD_4	ND	ND
LTE_4	ND	ND
RvD_1	ND	ND
RvD_2	ND	ND
RvD_3	ND	ND
RvD_5	ND	ND
RvE_1	ND	ND
TXB_2	ND	ND
NPD_1	ND	ND
Maresin-1	ND	ND
Maresin-2	ND	ND

Supplementary Table 2: Rodent ocular angle tissue cytokine analysis results with and without lipoxin A_4 treatment (*indicates a significant difference between vehicle and LXA_4 groups).

**Analyte**	**Vehicle (pg/mL)**	**LXA_4 (pg/mL)**	**Analyte**	**Vehicle (pg/mL)**	**LXA_4 (pg/mL)**
TGF-β_1	23.16±2.27	27.98±2.57	IL-6	330.65±30.95	303.18±28.80
TGF-β_2	123.68±11.89	134.07±8.44	IL-10	13.34±0.84	12.82±1.28
**TGF-β_3***	**9.78±1.17**	**15.79±1.98**	**IL-12p70***	**12.60±1.68**	**5.73±1.54**
EGF	7.77±1.61	5.99±2.06	IL-13	6.90±1.21	5.53±0.80
Eotaxin	2.92±0.26	2.86±0.25	IL-17A	2.37±0.24	2.56±0.36
Fractalkine	132.57±14.13	125.14±11.08	IL-18	3954.39±329.4	3452.56±209.04
G-CSF	2.07±0.20	2.61±0.34	IP-10	11.99±0.80	11.82±1.32
GM-CSF	10.627±2.19	13±2.16	Leptin	257.24±35.01	194.41±19.57
GRO/KC	35.987±8.86	59.03±6.13	LIX	17.67±0.95	14.65±1.23
IFN-γ	114.15±4.83	104.44±6.27	MCP-1	55.68±11.32	76.79±11.72
IL-1α	21.40±5.37	14.88±2.11	**MIP-1α***	**3.41±0.26**	**2.75±0.16**
IL-1β	45.71±3.56	44.24±3.21	MIP-2	11.25±1.88	11.67±3.32
IL-2	12.64±1.69	9.23±1.25	RANTES	10.56±1.38	12.76±1.57
IL-4	9.55±1.45	9.53±1.32	**TNF-α***	**7.55±0.95**	**4.12±0.60**
IL-5	22.65±1.64	19.91±0.97	VEGF	30.64±2.45	33.69±1.59

Supplementary Figure 1

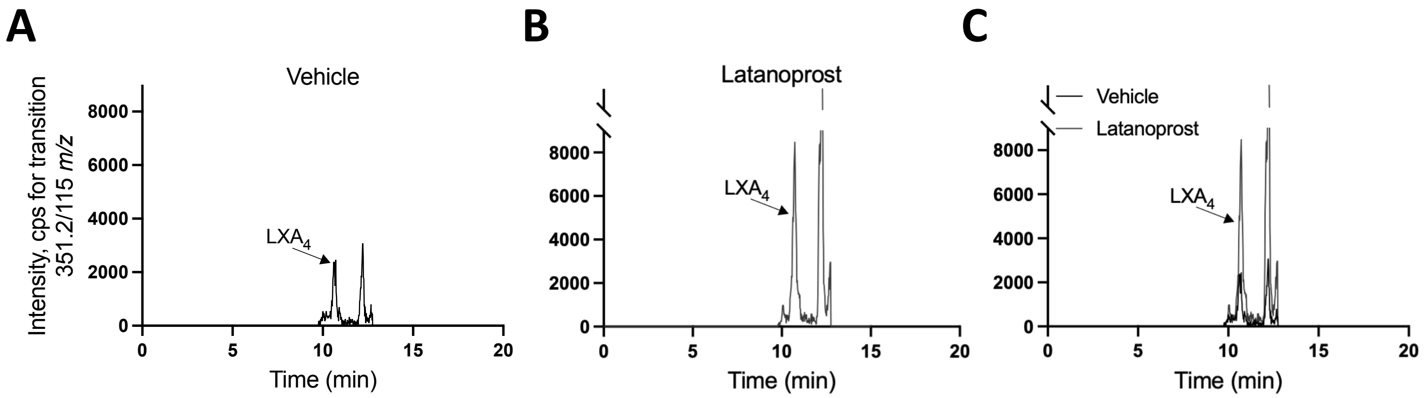

Supplementary Figure 1. Representative LC-MS/MS analyses in negative ion mode using scheduled Multiple Reaction Monitoring (MRM). Extracted ion chromatogram (XIC) showing the intensity, counts per second (cps) for LXA_4 transition 351.2/115 *m/z* on the Y-axis and retention time on the X-axis for **A)** Vehicle (black), **B)** Latanoprost 50µm (blue), **C)** Combined graph for vehicle (black) and latanoprost 50µm (blue). The arrow indicates the LXA_4 peak, which matches the retention time for LXA_4 in external standards.

.

Supplementary Figure 2

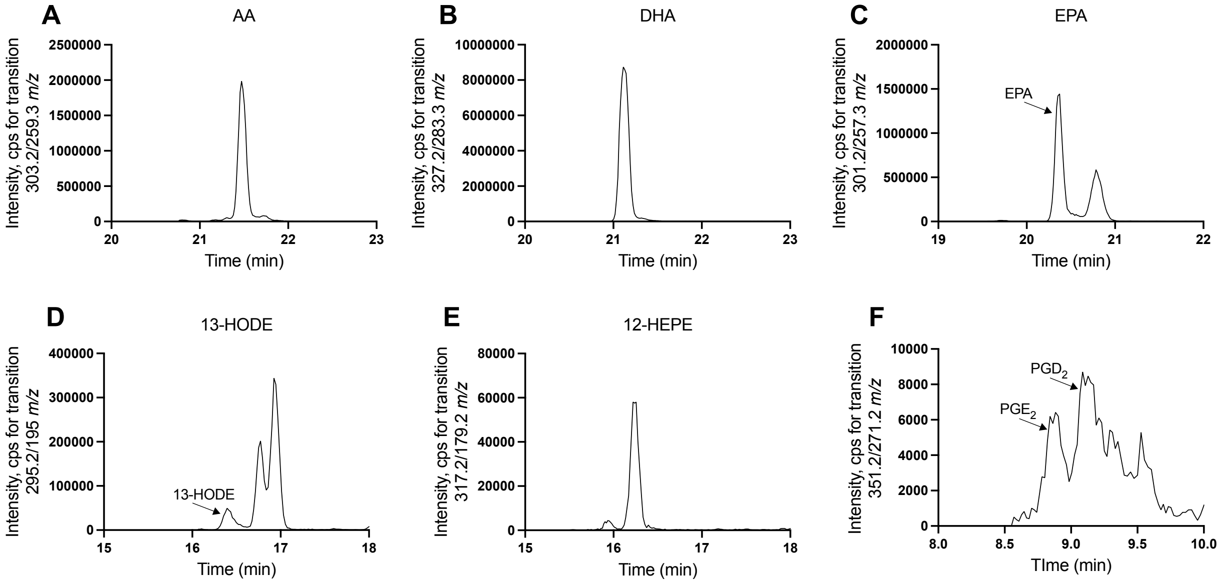

Supplementary Figure 2. LC-MS/MS analyses of Latanoprost (50μm) treated TM cells in negative ion mode using scheduled Multiple Reaction Monitoring (MRM) for additional analytes. Extracted ion chromatogram (XIC) showing the intensity, counts per second (cps) for *m/z* transitions on the Y-axis and retention time on the X-axis for **A)** AA- 303.2/259.3 *m/z*, **B)** DHA- 327.2/283.3 *m/z*, **C)** EPA- 301.2/257.3 *m/z*, **D)** 13-HODE- 295.2/195 *m/z*, **E)** 12-HEPE- 317.2/179.2 *m/z*, **F)** PGs- 351.2/271.2 *m/z*. The arrow indicates marked peaks, which matches the retention time for respective external standards. AA- Arachidonic acid, DHA- Docosahexaenoic acid, EPA- Eicosapentaenoic acid, 13-HODE- 13-hydroxyoctadecadienoic acid, 12-HEPE- 12-hydroxyeicosapentaenoic acid, PG- Prostaglandins.

Supplementary Figure 3

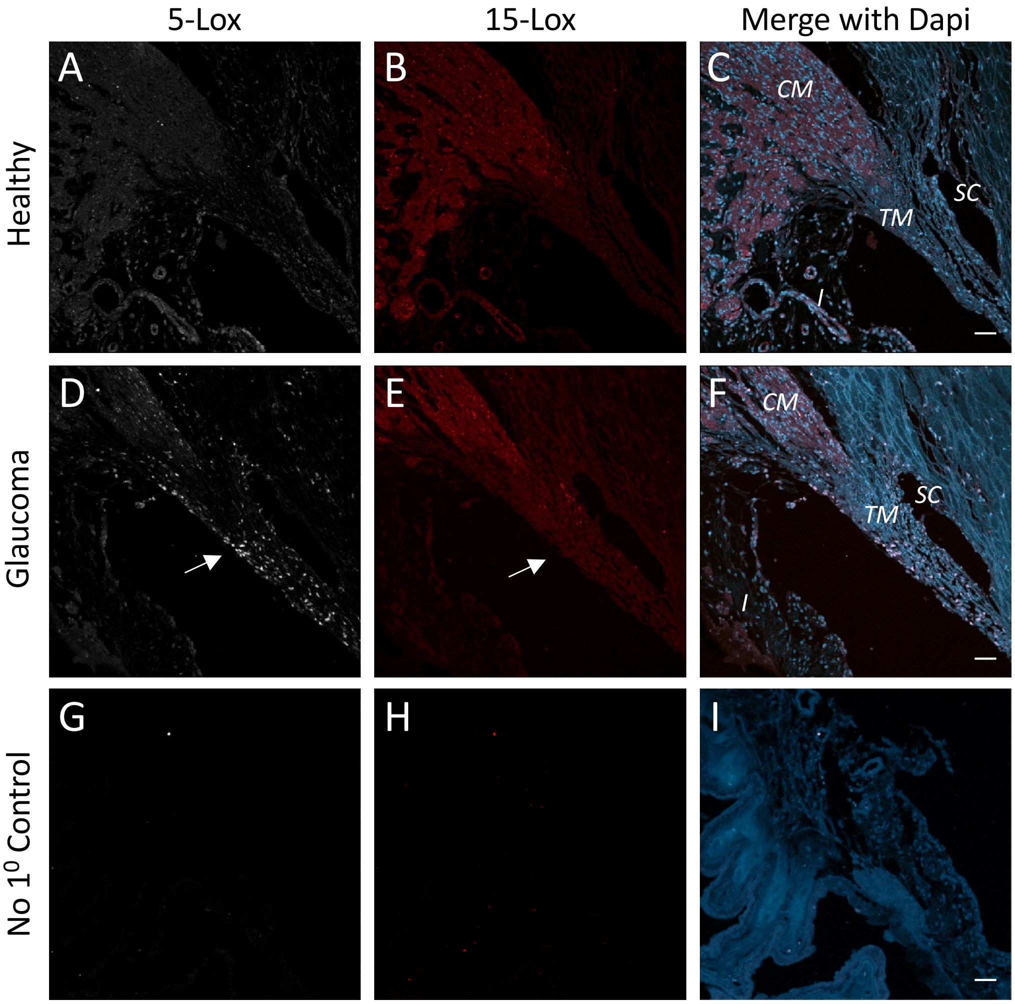

Supplementary Figure 3. 5- and 15-LOX staining of human anterior segment tissues. (A-C) Representative sections from a healthy human eye, stained with antibodies directed to 5-LOX (white) and 15-LOX (red), highlight the ciliary muscle (*CM*), iris (*I*) and associated vasculature, and outflow tissues, including the trabecular meshwork (*TM*) and Schlemm's canal (*SC*). **(D-F)** Representative sections from a human glaucomatous eye showing increased 5-LOX, and slightly increased 15-LOX in the *TM* (arrows). **(G-I)** Control sections without primary antibody staining were generally blank. (Scale bars represent 50 μm).

Supplementary Figure 4

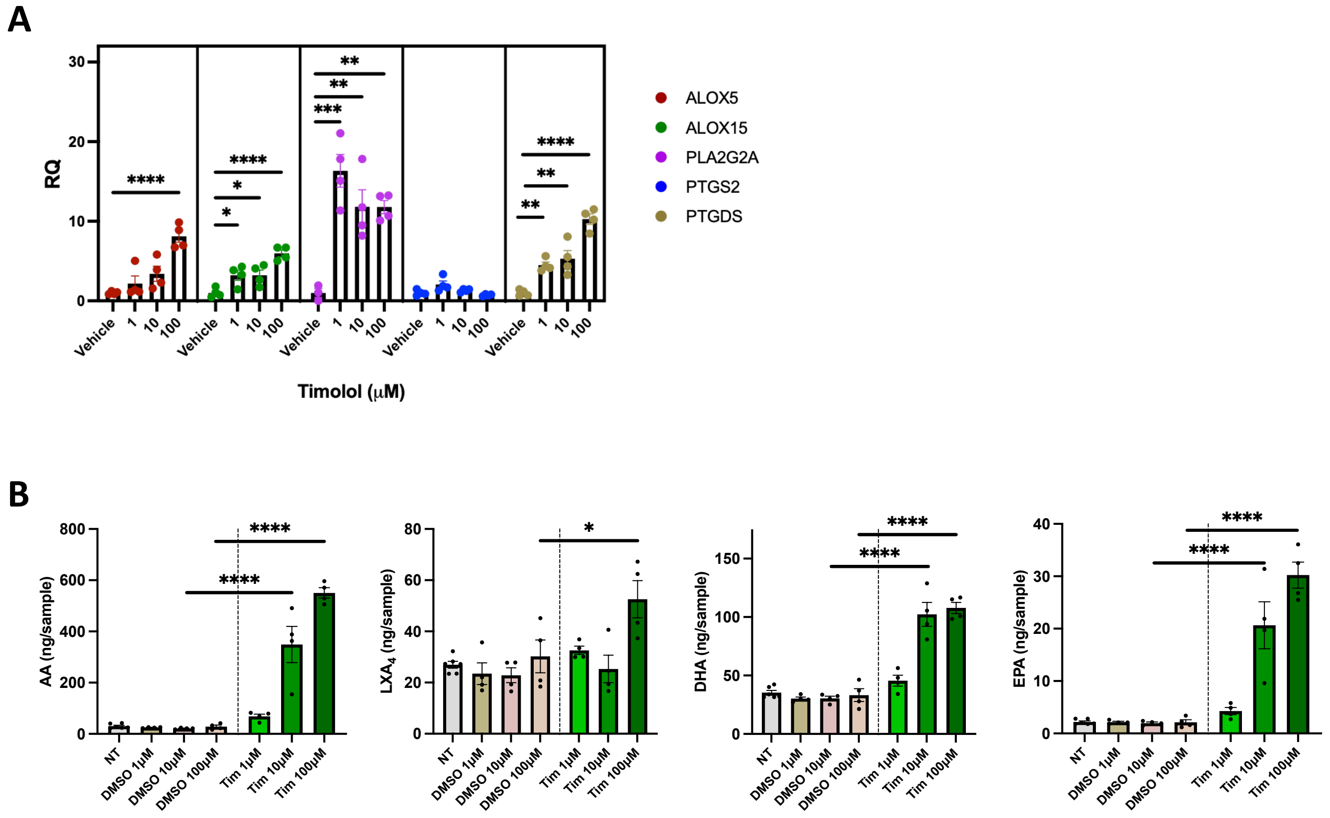

Supplementary Figure 4. Key lipid mediator synthetic enzymes and synthesis were induced by timolol. (**A**) Treatment with increasing concentrations of timolol significantly upregulated expression of ALOX5, ALOX15, PLA2G2A and PTGDS. (**B**) Lipidomic analyses of the timolol treated culture media showed a significant, dose dependent increase in arachidonic acid and LXA_4 levels, as well as DHA and EPA substrates. (*p<0.05, **p<0.01, ***p<0.005, ****p<0.001, bars are SE).

Supplementary Figure 5

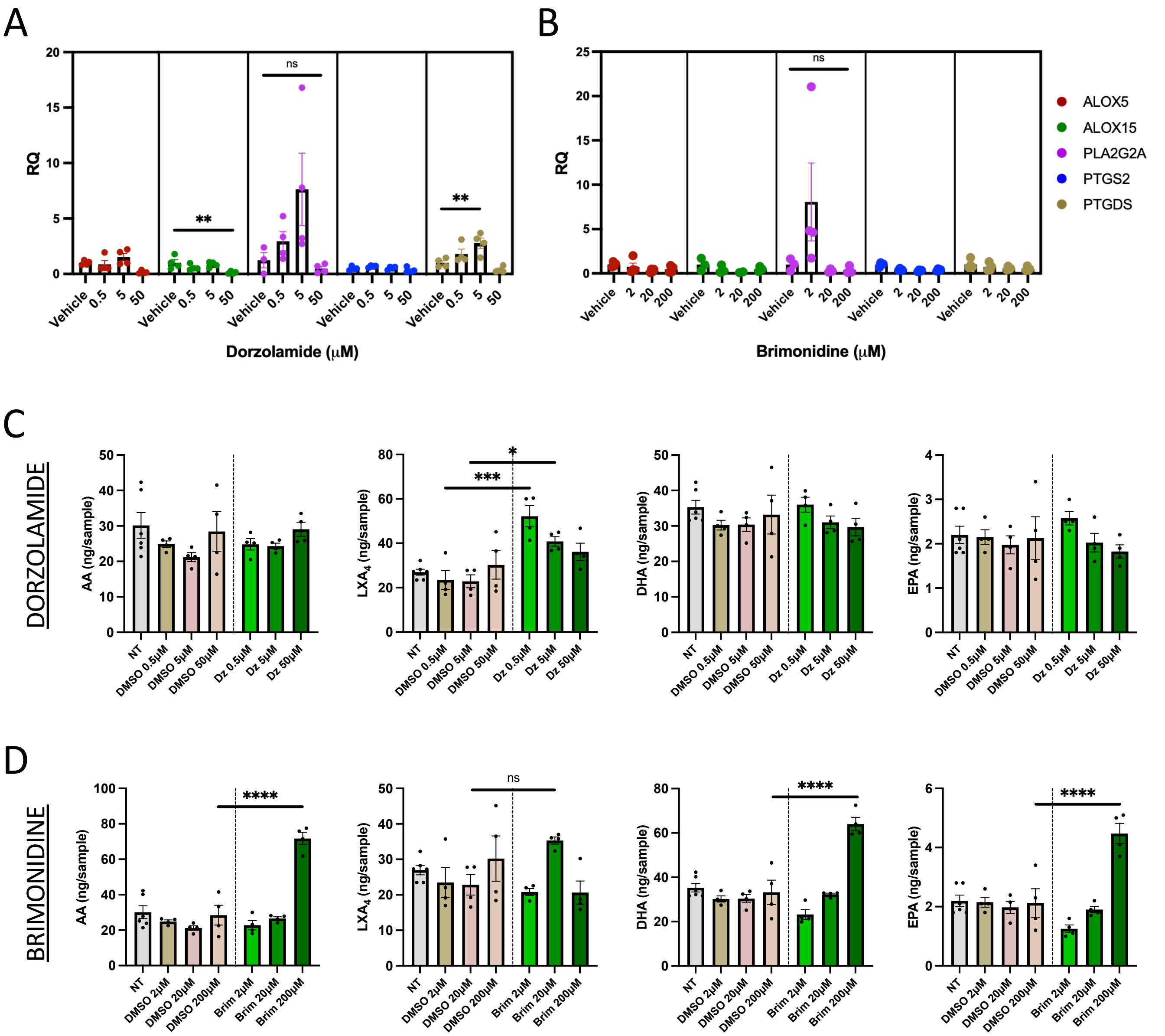

Supplementary Figure 5. Key lipid mediator synthetic enzymes and synthesis were not strongly affected by dorzolamide or brimonidine. (**A-B**) In contrast to Latanoprost and Timolol, Dorzolamide treatment had little effect on a panel of key synthetic enzymes, resulting in downregulation of all transcripts at 50 μm (A). Similarly, Brimonidine treatment did not result in any significant changes (B). (**C-D**) Concentrations of PUFA precursors and LXA_4 from analyses of TM cell culture media following treatment with dorzolamide or brimonidine. (*p<0.05, **p<0.01, ***p<0.005, ****p<0.001, bars are SE).

Supplementary Figure 6

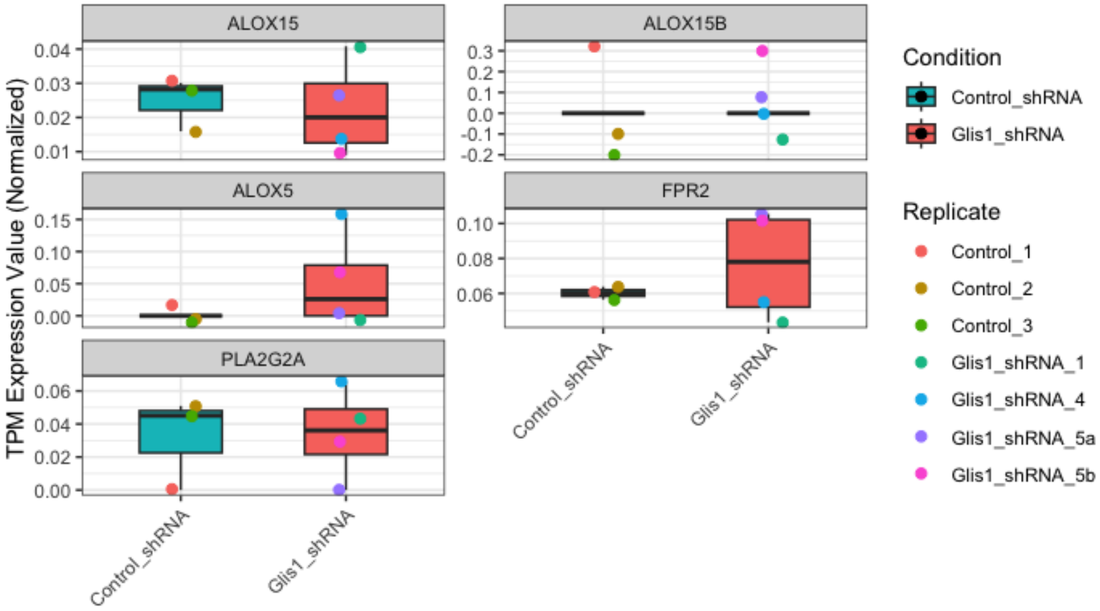

Supplementary Figure 6. Analyses of LXA_4 synthetic pathway gene expression in HTM cells. HTM cell RNAseq data were mined from Nair et al, 2021 (PMID: 34385434, GSE: GSE156846). The data compares normalized expression of synthetic LXA_4 genes and FPR2 in controls vs cells containing a knockdown for *Glis1 (Glis1_shRNA);* a gene linked to TM dysfunction and POAG. No substantive differences were identified.

Supplementary Figure 7

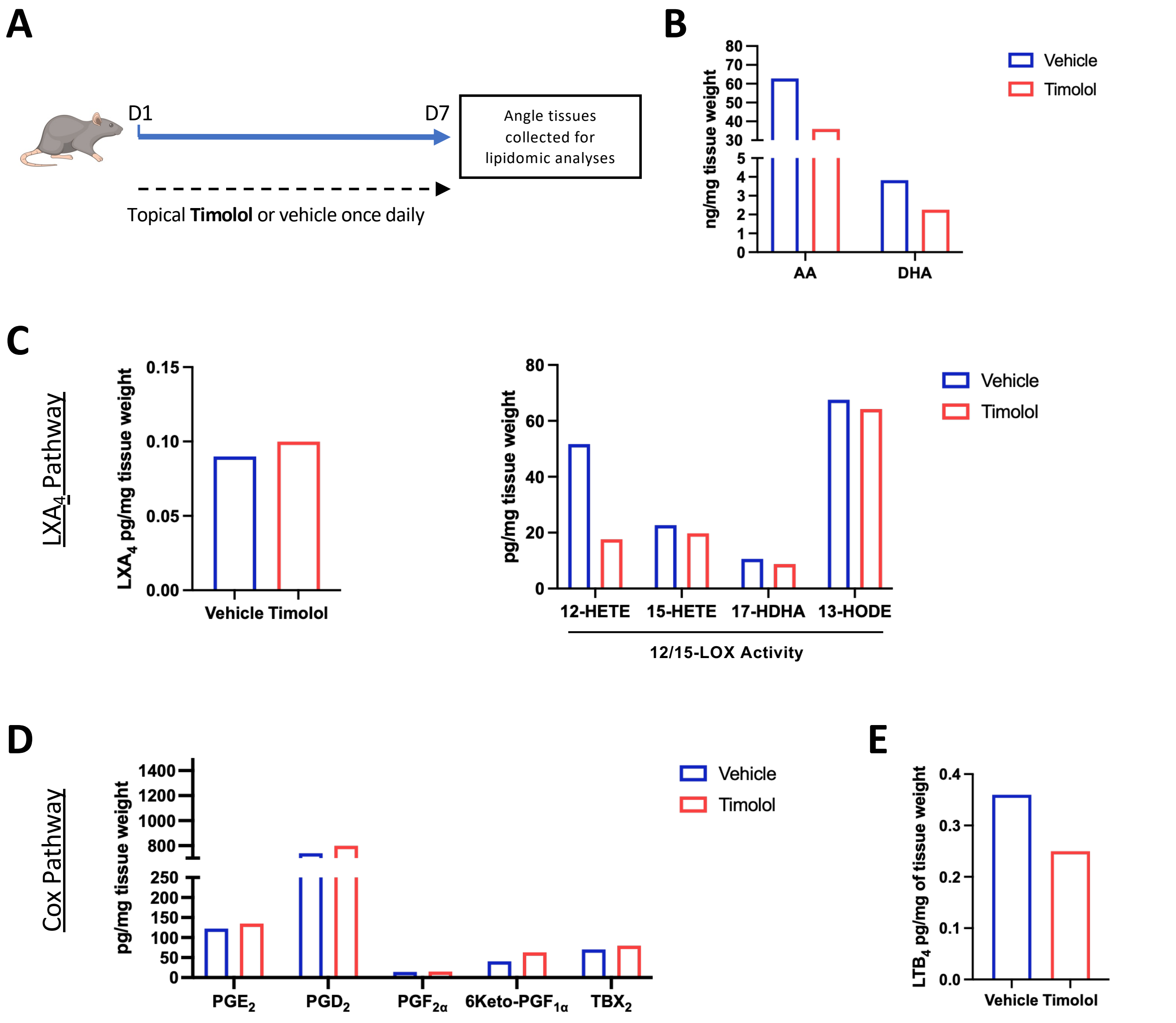

Supplementary Figure 7. Analyses of LXA_4 and COX pathway mediators after treatment with timolol shows no induction *in vivo*. A) Rats were dosed topically with timolol or vehicle daily for 7 days and angle tissues were collected and pooled for lipidomic analyses. B) Concentrations of PUFAs detected in angle tissues. C) Concentrations of products and intermediates in the LXA_4 pathway. D) Concentrations of Cox pathway products. E) Concentration of LTB4.
